# A Noise-Robust, Baseline-Free, and Adaptive Damage Indicator of Plate-like Structures Based on the Multicomponent Information Separation of High-Resolution Mode Shapes Using Wavelets

**DOI:** 10.3390/s25092669

**Published:** 2025-04-23

**Authors:** Yuexin Wang, Tongfa Deng, Jinwen Huang, Maosen Cao, Dragoslav Sumarac

**Affiliations:** 1 School of Civil and Surveying & Mapping Engineering, Jiangxi University of Science and Technology, Ganzhou 341000, China; 6120220347@mail.jxust.edu.cn (Y.W.); hjwshm@hhu.edu.cn (J.H.); cmszhy@hhu.edu.cn (M.C.); dsumarac@np.ac.rs (D.S.); 2 Jiangxi Province Key Laboratory of Environmental Geotechnical Engineering and Hazards Control, Jiangxi University of Science and Technology, Ganzhou 341000, China; 3 College of Mechanics and Engineering Science, Hohai University, Nanjing 211100, China; 4 Department of Technical Sciences, Civil Engineering, State University of Novi Pazar, 36300 Novi Pazar, Serbia

**Keywords:** damage identification, mode shape, noise robustness, wavelet transform, digital image correlation

## Abstract

Modal-based damage identification methods, particularly those utilizing mode shapes, excel in damage localization due to their spatial distribution characteristics. Traditional contact-based sensors often struggle with achieving high spatial resolution, while optical dynamic measurements, though high-resolution, are susceptible to environmental noise. This study proposes a novel multicomponent information separation (MIS) approach to overcome these challenges. The method decomposes mode shape information into noise, damage-related features, and waveform trends. The methodology begins with optical dynamic measurement to capture high-resolution mode shapes, followed by two-dimensional continuous wavelet transform (2D-CWT) for multiscale analysis. Adaptive wavelet scale selection via scale correlation analysis (SCA) improves noise separation, and an iterative weighted least squares fitting (IWLSF) method accurately fits the waveform trends. Finally, a noise-robust and baseline-free damage indicator (DI) is constructed through a difference operation to eliminate waveform trends, with damage localized at DI abrupt change positions. Validated through numerical simulations and experiments on damaged aluminum plates, the method demonstrates superior noise robustness and localization accuracy for multiple minor damages compared to existing wavelet-based approaches, showcasing its potential for advancing damage detection techniques.

## 1. Introduction

Identifying early damage to structures during service plays a vital role in maintaining structural integrity and safety. In recent decades, modal-based dynamic damage identification has developed rapidly and attracted increasing attention due to its advantages of global detection and having no influence on the normal use of structures [1,2,3,4]. Modal-based damage identification methods mainly identify damage by comparing the change in modal parameters (frequency, damping, and mode shape) or modal derivatives before and after damage [5].

According to different modal parameter categories, dynamic damage identification methods can be divided into the following three categories [6]: (1) Frequency-based dynamic damage identification [7,8,9]: it is easier to obtain the natural frequency and it is the earliest modal parameter used for damage detection [10]. (2) Damping-based dynamic damage identification [11,12]: There is less research on damage identification using damping ratios. On the one hand, in practical engineering, the measurement of damping has poor stability. Damping results are different according to different measurement methods [13,14]. On the other hand, damping is a parameter reflecting the global dynamic characteristics of a structure. It does not contain spatial information about damage. Therefore, it is usually combined with other theories or algorithms to realize damage identification. (3) Dynamic damage identification based on mode shapes and their derivatives: Mode shape and its derivatives are the most important parameters for damage identification, especially for damage localization [15]. Because the spatial information of mode shapes containing damage information can reflect the local dynamic characteristics of structures, a series of associated methods have been developed [16]. Moreover, there are many derivatives of mode shape, and the sensitivity of these derivatives to damage is often higher than that of mode shapes [17].

Numerous studies have investigated dynamic damage identification based on mode shapes and their derivatives. For example, mode shapes [18], mode shape curvatures [17,19], modal strain energy [20], and flexibility [21] have been used widely. Hassani et al. [22] pointed out that mode shape is sensitive to small changes in the system. Therefore, they proposed a two-stage method for damage detection in structures with closely spaced eigenvalues based on mode shape sensitivity. Yang et al. [23] proposed a movable sensor system to measure the mode shape of a simply supported bridge. They pointed out that the reconstruction of mode shape is important for damage detection since it contains location information.

Traditional contact measurement technologies, such as sensors and strain gauges [24], are limited in their ability to acquire dense response data due to measurement point arrangement constraints. Consequently, non-contact measurement techniques have emerged. For plate-like structures, non-contact measurement enables the acquisition of full-field response data [25]. Shi et al. [26] proposed a multi-mode shape fusion approach to identify line-type damage in plate-type structures. They obtained a high-resolution mode shape using an excitation–response system of Piezoelectric Leadzirconate–Titanate (PZT) and Scanning Laser Doppler Vibrometers (SLDVs). However, the acquisition of high-resolution mode shapes is susceptible to noise [27]. To address this, Xu et al. [28] defined a noise-robust damage index for carbon-fiber-reinforced polymer (CFRP) laminates. By integrating multi-resolution analysis and the Teager–Kaiser energy operator, their index based on high-resolution mode shape exhibited high damage sensitivity and strong noise resistance. Sun et al. [29] discussed the influence of the number of degrees of freedom (DOFs) in the measurement damage identification. They pointed out that a non-contact optical dynamic measurement system using a high-speed camera exhibits a compelling capacity for nondestructive damage detection.

For the extraction of singularity features from high-resolution mode shapes, wavelet analysis proves to be a robust analytical tool. To improve two-dimensional curvature mode shape (CMS^2D^), Xu et al. [30] introduced wavelet transform and the TEO energy operator to improve CMS^2D^, proposing the damage index TEO-WT CMS^2D^. Sun et al. [29] proposed an index, *TDI_wf_*, for damage identification through the fusion of multiple scales of wavelet coefficients. It can be seen from the existing research that wavelets are effective in extracting damage features from mode shapes, but there is no discussion of scale selection for wavelets in the existing research.

Based on the above analysis, the practical application of damage identification methods for plate-like structures remains constrained by several limitations: (1) The sparse response data from limited measurement points seriously reduces the damage location accuracy of dynamic damage identification methods. (2) Not only structural damage can cause structural dynamic characteristic parameters to change; environmental noise can also cause them to obviously change. However, the existing dynamic damage indicators are not able to resist noise, especially those based on high-resolution mode shape. (3) The effectiveness of wavelet-based damage identification is largely dependent on the determination of wavelet transform scales; nevertheless, quantitative foundations for scale selection are currently lacking in the literature. (4) The prevalence of multiple damage scenarios in practical applications underscores the need for damage indices capable of identifying multiple simultaneous damages.

To address these issues, this work proposes a noise-robust, baseline-free, adaptive DI based on the MIS of high-resolution mode shapes for plate-like structures. To achieve dense measurement-point distribution and obtain high-resolution dynamic response data, this work implements a non-contact optical dynamic measurement system based on high-speed camera technology. This approach demonstrates superior efficiency in acquiring full-field responses compared to the traditional point-by-point scanning method using laser vibrometers. Measurement accuracy and computational efficiency are substantially enhanced through an integrated approach [27] combining a single accelerometer with high-speed camera measurements and Welch power spectral density (PSD) estimation. On the theoretical front, this work employs a comprehensive framework incorporating two-dimensional continuous wavelet transform (2D-CWT), iterative weighted least-squares fitting (IWLSF), and signal correlation analysis (SCA). Based on these theories, a comprehensive multiscale analysis framework is developed to decompose information into three distinct scales: the fields of the noise, damage, and waveform trends of the structural dynamic response. This enables the identification of multiple sources of damage in plate-like structures under conditions of severe noise interference without requiring baseline data. To validate the improvement of the proposed method, comparative analyses are conducted against CMS^2D^ and two established wavelet-based damage indicators (TEO-WT CMS^2D^ and *TDI_wf_*).

The remainder of this paper is organized as follows. The proposed DI is presented in Section 2. To verify the effectiveness of the proposed DI, finite element models of cantilever plates with multiple damages are established by ANSYS 2020R2 in Section 3. The damage detection experiment for an artificially scratched aluminum plate is presented in Section 4 to verify the practical applicability of the method. Finally, our conclusions are summarized in Section 5.

## 2. Noise-Robust Adaptive DI

### 2.1. Strategy Framework of Damage Identification Based on MIS

**Figure 1:**
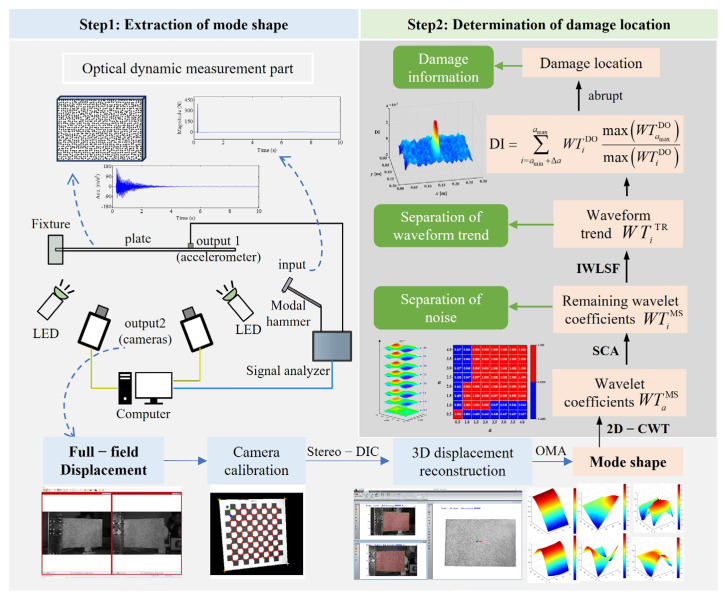
The framework of damage identification based on MIS.

This work presents a noise-robust, baseline-free, adaptive DI for plate-like structures using wavelets and optical measurement. Damage identification is performed in two steps: high-resolution mode shape extraction and subsequent noise and waveform trend elimination via MIS, as shown in Figure 1.

Step 1: Extraction of mode shape.

(1) Carry out the modal experiment for a plate-like structure by using high-speed cameras and one accelerometer;
(2) Reconstruct the full-field three-dimensional displacement of the structure by stereo digital image correlation (stereo-DIC);
(3) Extract high-resolution mode shapes from the response data through operating modal analysis (OMA).

**Figure 2:**
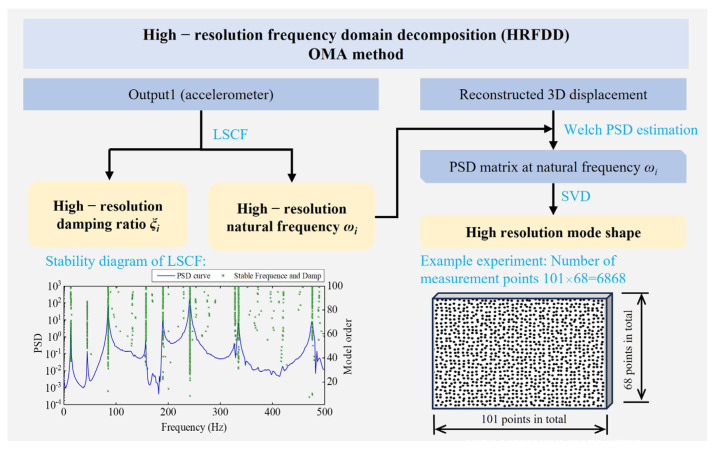
A flowchart of the OMA approach.

Due to the lack of a fully developed commercial DIC-based modal analysis package for plate-like structures, an appropriate technique for handling dense measurement points was developed, and the flowchart of the OMA approach is shown in Figure 2. Firstly, the Least Squares Complex Frequency Domain (LSCF) method is used to process the acceleration response and obtain the natural frequency and damping ratio. The LSCF method fits a right matrix fractional model (RMFM) to the PSD function as follows [31]:

(1) $$\left[G\left(\omega \right)\right]=\left[B\left(\omega \right)\right]{\left[A\left(\omega \right)\right]}^{-1}$$

where $G\left(\omega \right)\in C^{l\times t}$ is the theoretical PSD matrix, *l* is the number of outputs, *t* is the number of reference outputs, and $B\left(\omega \right)\in C^{l\times t}$ and $A\left(\omega \right)\in C^{t\times t}$ are matrix polynomials and the numerator and denominator of the RMFM, respectively. Secondly, Welch PSD estimation is applied to the reconstructed displacement. The PSD matrix is calculated directly at natural frequency for efficiency. The cross-PSD function ${\hat{G}}_{12}\left(\omega \right)$ of the response signals $x_{1}\left(t\right)$ and $x_{2}\left(t\right)$ is calculated as follows [32]:

(2) $${\hat{G}}_{12}\left(\omega \right)=\frac{1}{N_{b}}\sum_{k=1}^{N_{b}}\left\{\frac{\sum_{t=1}^{M}w\left(t\right)x_{1}\left(t\right)e^{-j\omega t}}{MP}\cdot conj\left[\sum_{t=1}^{M}w\left(t\right)x_{2}\left(t\right)e^{-j\omega t}\right]\right\}$$

where ω is the circular frequency, $N_{b}$ is the total number of segments dividing the response signal, k represents the k-th segment signal, M is the length of each segment of data, $w\left(t\right)$ is a non-rectangular window function applied to reduce energy leakage, $conj\left(\cdot \right)$ denotes a conjugate operation, and P denotes a normalization factor, which can be expressed as follows:

(3) $$P=\frac{1}{M}\sum_{t=1}^{M}{\left|w\left(t\right)\right|}^{2}$$

The mode shapes are obtained by performing singular value decomposition on the PSD function at natural frequencies. For details of this method, please refer to reference [27].

Step 2: Determination of damage location.

(1) Apply 2D-CWT to obtain wavelet coefficients at different scales;
(2) Separate the noise of the obtained mode shape according to the SCA;
(3) Separate the waveform trend of the denoised mode shape according to the IWLSF;
(4) Construct the DI for damage identification.

### 2.2. The Information of Mode Shape

In the existing study, as the derivative of mode shape, two-dimensional curvature mode shape (CMS^2D^) can be used to detect the damage because of the ability inheriting from the mode shape that there is abundant spatial information in the mode shape. Information about the mode shape can be observed in the CMS^2D^. It can be calculated by performing a second-order central difference operation on the mode shape, as follows [30]:

(4) $${\mathrm{CMS}}^{2D}=\kappa_{xx}+\kappa_{yy}=\frac{\partial^{2}W\left(x,y\right)}{\partial x^{2}}+\frac{\partial^{2}W\left(x,y\right)}{\partial y^{2}}$$

where $\kappa_{xx}$ and $\kappa_{yy}$ are CMS^2D^ in the x and y directions, respectively.

According to the vibration theory for thin plates, the bending moments $M_{x}\left(x,y\right)$ and $M_{y}\left(x,y\right)$ of a thin plate can be written as follows:

(5) $$M_{x}\left(x,y\right)=-D\left(x,y\right)\left(\frac{\partial^{2}W\left(x,y\right)}{\partial x^{2}}+\upsilon \frac{\partial^{2}W\left(x,y\right)}{\partial y^{2}}\right)$$

(6) $$M_{y}\left(x,y\right)=-D\left(x,y\right)\left(\frac{\partial^{2}W\left(x,y\right)}{\partial y^{2}}+\upsilon \frac{\partial^{2}W\left(x,y\right)}{\partial x^{2}}\right)$$

where $D\left(x,y\right)$ is the bending stiffness of a thin plate. For homogeneous plates, the bending stiffness $D\left(x,y\right)=\left(Eh^{3}/12\left(1-\upsilon^{2}\right)\right)$, where υ is Poisson’s ratio, E is the elastic modulus, and *h* is the thickness of the thin plate. Substituting Equations (5) and (6) into Equation (4) yields the following equation:

(7) $${\mathrm{CMS}}^{2D}=\frac{\partial^{2}W\left(x,y\right)}{\partial x^{2}}+\frac{\partial^{2}W\left(x,y\right)}{\partial y^{2}}=\frac{-M_{x}\left(x,y\right)-M_{y}\left(x,y\right)}{D\left(x,y\right)\left(1+\upsilon \right)}$$

**Figure 3:**
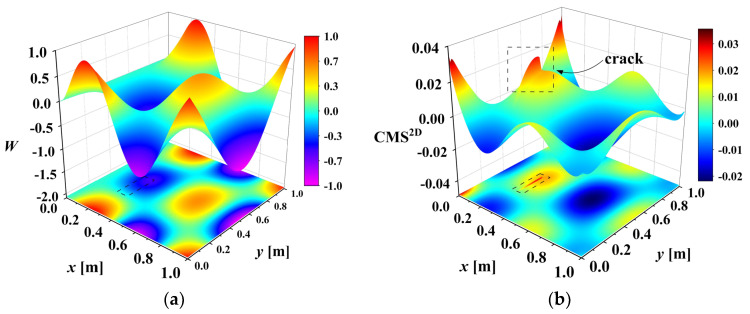
Crack localization based on CMS^2D^: (**a**) 10th order mode shape *W* of cracked cantilever plate; (**b**) CMS^2D^ of damaged plate.

Damage will reduce the local bending stiffness $D\left(x,y\right)$. According to Equation (1), a change in $D\left(x,y\right)$ at the damage location will lead to a change in CMS^2D^. In the left half of Figure 3, *W* represents the 10th-order mode shape of a cracked cantilever plate. The crack position, indiscernible in the mode shape, is readily identified by the abrupt change in CMS^2D^.

For plate-like structures, mode shape has an outstanding potential for damage identification because of its abundant spatial information. The strategy proposed in this work examines mode shapes from global information at large scales to local details at small scales using multiscale analysis. This is performed from the perspective of extracting damage information from the mode shape through MIS; studying the correlation between the scales by SCA; and eliminating waveform trends in the chosen scales by IWLSF. In brief, the main idea of the MIS is to highlight damage information and weaken the interference of noise and waveform trends.

### 2.3. Multiscale Analysis of Mode Shape

In the field of signal processing, wavelet transform is a commonly used multiscale analysis tool. It can focus on any detail of a signal to be processed flexibly using expansion and translation transformations and effectively describe the local characteristics of a signal. Therefore, wavelet transform is known as a “signal microscope”. When wavelet analysis is applied to modal data, the wavelet basis function should be selected according to the dimension of the mode shape. In the mode shape analysis of beams and columns, 1D-CWT is typically employed due to the one-dimensional nature of the data. For plates and shells, 2D-CWT is used because the data are two-dimensional. In this paper, damage identification methods for plate-type structures are studied, and 2D-CWT is introduced below.

For a two-dimensional signal $f\left(x\right)\in L^{2}\left(R^{2}\right)$, its 2D-CWT can be expressed as the following Equation [28]:

(8) $$WT\left(b,a,\theta \right)=a^{-1}\int_{R^{2}}f\left(x\right)\cdot \psi^{*}\left(a^{-1}r_{-\theta }\left(x-b\right)\right)d^{2}x$$

where WT represents the transformed wavelet coefficients, ${\left[•\right]}^{*}$ represents the complex conjugate operation, *a* is the scale factor, *b* is the translation factor, θ is the rotation factor, $\psi \left(x\right)$ represents the mother wavelet function, and $r_{-\theta }$ is the rotation matrix determined by θ, which can be expressed as follows:

(9) $$r_{-\theta }=\left[\begin{array}{cc} \cos\left(\theta \right) & -\sin\left(\theta \right)\\ \sin\left(\theta \right) & \cos\left(\theta \right) \end{array}\right]$$

Equation (8) can also be expressed in Fourier transform form:

(10) $$WT\left(b,a,\theta \right)=a^{-1}\int_{R^{2}}\hat{f}\left(x\right)\cdot {\hat{\psi }}^{*}\left(a^{-1}r_{-\theta }\left(\omega \right)\right)e^{ib\cdot \omega }d^{2}\omega$$

where $\hat{\left[•\right]}$ represents Fourier transform and $\omega =\left(\omega_{x},\omega_{y}\right)$ is spatial frequency.

In this work, the Mexican-hat wavelet is chosen as the optimal mother wavelet function. The Mexican-hat wavelet has attractive properties, such as rotational invariance, appropriate vanishing moments, compactness, and symmetry, which give it a powerful ability to extract different kinds of damage features.

**Figure 4:**
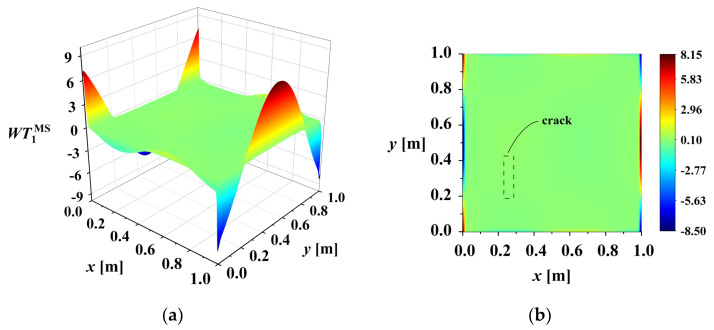
WT coefficient map of mode shape of plate with crack damage (scale = 1): (**a**) $WT_{1}^{\mathrm{MS}}$; (**b**) X-Y view of $WT_{1}^{\mathrm{MS}}$.

**Figure 5:**
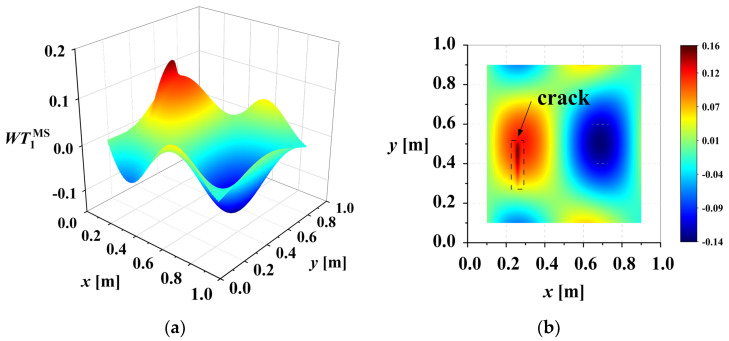
WT coefficient map of mode shape of plate with crack damage after removing boundary coefficients (scale = 1): (**a**) $WT_{1}^{\mathrm{MS}}$; (**b**) X-Y view of $WT_{1}^{\mathrm{MS}}$.

A rotation-invariant mother wavelet is chosen, so the rotation factor θ is ignored in this study. The 2D-CWT of the mode shape (MS) is defined as $WT_{a}^{\mathrm{MS}}\left(b\right)$. It is noticed that there is boundary distortion in the wavelet transform. For example, Figure 4 shows the *WT* coefficient maps of the mode shape in Figure 3a at scale *a* = 1. The figure reveals abrupt changes in wavelet coefficients at the boundary. These boundary effects can obscure damage-induced changes. Removing the boundary coefficient values, as shown in Figure 5, clearly reveals the abrupt peak associated with the damage.

**Figure 6:**
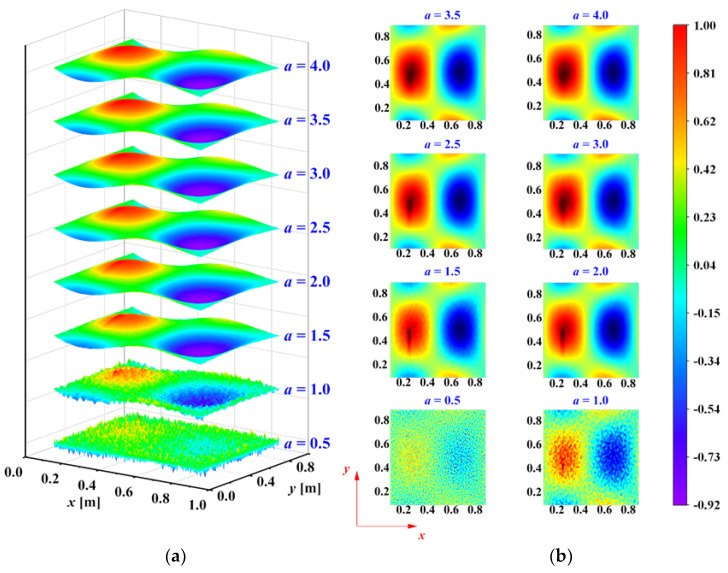
WT coefficient maps of mode shapes of plate with crack damage after removing boundary coefficients at different scales (scale = 0.5, 1.0, …, 4.0): (**a**) $WT_{a}^{\mathrm{MS}}$; (**b**) X-Y view of $WT_{a}^{\mathrm{MS}}$.

Besides the mother wavelet, the decomposition scale is another crucial parameter in damage identification. The studies [33,34,35] have shown that different decomposition scales reveal different information components within the mode shapes of damaged structures. Noise is dominant at small scales, while waveform trends dominate at large scales. Damage information, in the form of singular features, is primarily found at intermediate scales. Thus, selecting an appropriate decomposition scale allows for the separation of noise, damage, and trend information. Consider the mode shape in Figure 3a. After adding noise and performing wavelet transform, the normalized $WT_{a}^{\mathrm{MS}}$ at different scales is shown in Figure 6.

In Figure 6, the damage information is basically submerged by noise at scales *a* = 0.5 and *a* = 1. These small scales are dominated by noise components. At larger scales, *a* = 3.0, *a* = 3.5, and *a* = 4.0, the $WT_{a}^{\mathrm{MS}}$ primarily reflects the waveform trend. For the intermediate scales *a* = 1.5, *a* = 2, and *a* = 2.5, the $WT_{a}^{\mathrm{MS}}$ provides the clearest indication of damage as the singular information is more prominent. The damage location can be estimated from the $WT_{a}^{\mathrm{MS}}$ values at these intermediate scales.

In conclusion, by selecting an appropriate mother wavelet function and decomposition scale, the CWT of mode shapes can effectively reveal the spatial location information of structural damage. This demonstrates the potential of CWT-based mode shapes for constructing noise-robust damage identification indices.

### 2.4. Damage Localization Indicator Based on MIS

According to the above analysis, in order to improve the mode-shape-based damage identification method using wavelets, SCA was introduced for scale selection research and a strategy for eliminating the waveform trend was developed by subtracting the fitting waveform trend obtained by IWLSF from $WT_{a}^{\mathrm{MS}}$.

#### 2.4.1. Signal Correlation Analysis (SCA)

The correlation coefficient *R* is usually used to measure the degree of correlation between two signals in SCA. Given two signals x(n) and $y\left(n\right)$, the correlation coefficient *R* between them is expressed through the following equation [36]:

(11) $$R=\frac{\sum_{i=1}^{N}\left(x\left(i\right)-\bar{x}\right)\left(y\left(i\right)-\bar{y}\right)}{\sqrt{\sum_{i=1}^{N}{\left(x\left(i\right)-\bar{x}\right)}^{2}}\sqrt{\sum_{i=1}^{N}{\left(y\left(i\right)-\bar{y}\right)}^{2}}}$$

where $\bar{x}$ and $\bar{y}$ are averages of x(n) and $y\left(n\right)$, respectively. *N* is the length of the signal, and the larger the $\left|R\right|$ is, the higher the correlation of the signals.

White noise and heavily noise-polluted signals can be identified and removed using SCA. These noisy signals exhibit very low correlations with other signals, approaching zero. Thus, these signals can be identified and removed based on their low correlation coefficients. After a lot of tests, we adopted a threshold value for $R_{\eta }$ of 0.95 in this study. The signals corresponding to scales whose correlation coefficients with other scales signals are all below the threshold will be eliminated.

However, for signals with similar trends, the correlation coefficient $\left|R\right|$ remains relatively high. When $\left|R\right|$ is higher than $R_{\eta }$, the waveform is considered to have covered the singular information of damage up, so this waveform signal should be eliminated.

#### 2.4.2. Iterative Weighted Least Squares Fitting (IWLSF)

**Figure 7:**
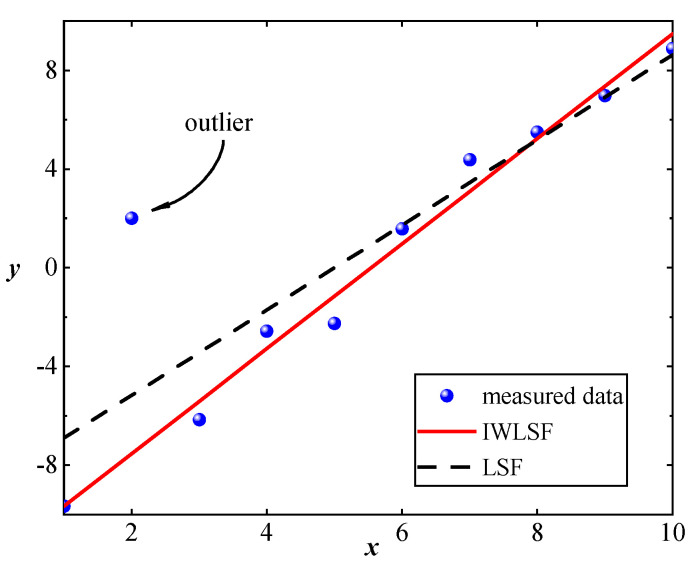
Comparison of LSF and IWLSF for straight-line fitting.

To eliminate waveform trend, the accurate fitting of the trend is significant. Ordinary least squares fitting (LSF) assigns equal weight to all the measured data, estimating parameters by minimizing the sum of the squared residuals. However, outliers in the measured data can significantly influence the fitting results, as LSF considers all data points. For straight-line fitting, it is clear that the LSF is not ideal due to outliers, as Figure 7 illustrates.

In damage identification, singularities at damage locations act as outliers in waveform trend fitting. Traditional LSF, sensitive to these outliers, produces a biased fitted trend, weakening damage features after trend removal. The IWLSF method was developed to weaken the influence of outliers. Unlike LSF, IWLSF uses a weighting function to assign each data point a weight coefficient. Points with large residual are assigned smaller weights. These weights are iteratively optimized to minimize the influence of outliers. The fitting objective function for IWLSF [37] is as follows:

(12) $$\sum_{i=1}^{n}w_{i}{\left(y_{i}-\varphi \left(x_{i}\right)\right)}^{2}=\min$$

where $y_{i}$ is the measured data, n is the total number of data points, φ(x) is the fitting model, and $w_{i}$ is the weight function. In this work, the weight function defined by Huber [38] is adopted, and its expression is as follows:

(13) $$w_{i}=\left\{\begin{array}{l} 1,\left|u_{i}\right|\leq c_{h}\\ \frac{c_{h}}{\left|u_{i}\right|},\left|u_{i}\right|>c_{h} \end{array}\right.$$

where $c_{h}$ is a constant with a value of 1.345; $u_{i}$ is the normalized residual of the *i*-th data point, which can be expressed as $u_{i}=e_{i}/s$, where $e_{i}$ is the residual of the *i*-th data point; and s is the residual scale factor, presented as follows:

(14) $$s=\frac{\mathrm{MAD}\left(e_{1},e_{2},\ldots ,e_{i}\right)}{0.6745}$$

(15) $$\mathrm{MAD}\left(e_{1},e_{2},\ldots ,e_{i}\right)=\mathrm{Median}\left|e_{i}-\mathrm{Median}\left(e_{1},e_{2},\ldots ,e_{i}\right)\right|$$

where Median is the median operator. The fitting procedure of IWLSF is as follows Algorithm 1:
**Algorithm 1:** Iterative weighted least squares fitting**Input:** Dataset data points **D****Output:** Fitting result vector
$\alpha^{\left(k\right)}$
1. Obtain the vector
$\alpha^{\left(1\right)}(\alpha_{1}^{\left(1\right)},\;\alpha_{2}^{\left(1\right)},\;\ldots ,\;\alpha_{m}^{\left(1\right)})$ by least squares fitting without weighting to **D**;  %$\alpha^{\left(1\right)}={\left(X^{T}X\right)}^{-1}X^{T}Y$XY are the independent and dependent variable matrixes, respectively).2. Calculate the normalized residual vector $u^{\left(k\right)}(u_{1}^{\left(k\right)},\;u_{2}^{\left(k\right)},\;\ldots ,\;u_{m}^{\left(k\right)})$;  %$u^{\left(k\right)}$ calculated through Equations (14) and (15).3. Calculate the weight coefficient vector $w^{\left(k\right)}\left(w_{1}^{\left(k\right)},\;w_{2}^{\left(k\right)},\;\ldots ,\;w_{m}^{\left(k\right)}\right)$;  $\%\;w^{\left(k\right)}$ calculated through Equation (13)4. Construct the diagonal matrix of the weight coefficient $W^{\left(k\right)}=diag\left(w^{\left(k\right)}\right)$.5. Obtain the vector
$\alpha_{1}^{\left(k\right)},\;\alpha_{2}^{\left(k\right)},\;\ldots ,\;\alpha_{m}^{\left(k\right)})$ by weighted LSF; %
$\alpha^{\left(k\right)}={\left(X^{T}WX\right)}^{-1}X^{T}WY$
6. Set the convergence threshold ε. When $\left|\alpha^{\left(k\right)}-\alpha^{\left(k-1\right)}\right|/\left|\alpha^{\left(k-1\right)}\right|\leq \varepsilon$, stop iteration.

It is worth noting that when using IWLSF, it is necessary to select a suitable fitting model φ(x). Straight-line fitting uses the following fitting model:

(16) $$\varphi \left(x\right)=kx+b$$

where *k* and *b* are the coefficients to be estimated. For the fitting of a waveform curve, it is appropriate to adopt a trigonometric polynomial for the fitting model φ(x), as follows:

(17) $$\varphi \left(x\right)=a_{0}+\sum_{i=1}^{n}a_{i}\cos\left(ix\right)+b_{i}\sin\left(ix\right)$$

where $a_{0}$, $a_{i}$, and $b_{i}$ are the coefficients to be estimated.

**Figure 8:**
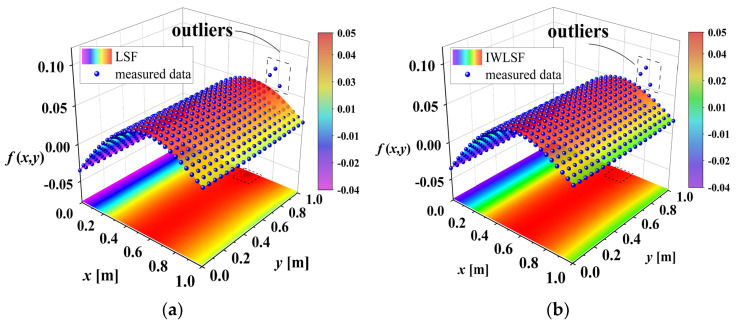
Comparison of LSF and IWLSF: (**a**) curved surface fitting of LSF; (**b**) curved surface fitting of IWLSF.

For waveform surface fitting, satisfactory fitting results can be obtained by using the following higher-order polynomial fitting model φ(x,y):

(18) $$\varphi \left(x,y\right)=\sum_{h=0}^{n_{1}}\sum_{s=0}^{n_{2}}c_{hs}x^{h}y^{s}$$

where $n_{1}$ and $n_{2}$ are the highest degrees of *x* and *y*, and $c_{hs}$ is the coefficient to be estimated. Figure 8 shows the curved surface fitting results of LSF and IWLSF, respectively. Equation (18) is adopted in the fitting process. As shown in Figure 8a, traditional LSF is sensitive to outliers, which can lead to the inaccurate fitting of the waveform trend. Consequently, using LSF to eliminate waveforms will weaken damage features. In contrast, Figure 8b demonstrates that IWLSF is less sensitive to outliers and has a higher fitting accuracy.

#### 2.4.3. Damage Localization Indicator

**Figure 9:**
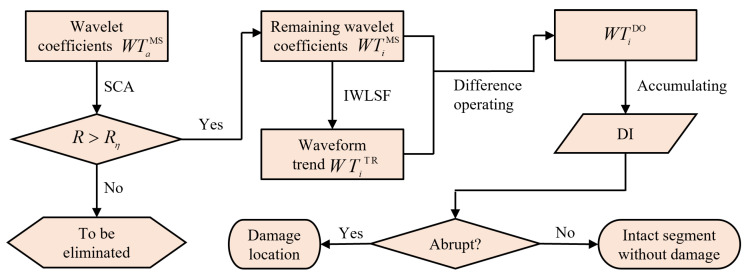
Flowchart of high-precision and noise-robust baseline-free damage identification method.

The procedure of damage identification is illustrated in Figure 9. Firstly, according to the weak correlation between white noise and other signals, SCA is used to identify and eliminate noise-dominated scales. Correlation analysis is carried out on the wavelet coefficients $WT_{a}^{\mathrm{MS}}$ at each scale, and each $WT_{a}^{\mathrm{MS}}$ with a correlation coefficient R lower than $R_{\eta }$ is eliminated. Although this reduces noise in the residual $WT_{i}^{\mathrm{MS}}$, the existence of a waveform trend can still affect damage localization. Subsequently, IWLSF fits the waveform trend surface $WT_{i}^{\mathrm{TR}}$ to $WT_{i}^{\mathrm{MS}}$. This trend $WT_{i}^{\mathrm{TR}}$ is then subtracted from $WT_{i}^{\mathrm{MS}}$. Finally, the noise-robust, baseline-free, and adaptive DI is constructed by accumulating the resulting scale coefficients, and its expression is as follows:

(19) $$\mathrm{DI}=\sum_{i=a_{\min}+\Delta a}^{a_{\max}}WT_{i}^{DO}\frac{\max\left(WT_{a_{\max}}^{DO}\right)}{\max\left(WT_{i}^{DO}\right)}$$

(20) $$WT_{i}^{DO}=WT_{i}^{MS}-WT_{i}^{TR}$$

(21) $$WT_{i}^{TR}=\mathrm{IWLSF}\left(WT_{i}^{MS}\right)$$

where Δa represents the scale step, $a_{\min}$ is the smallest scale after filtering out the noise scale by SCA, $WT_{i}^{DO}$ is the wavelet coefficient after eliminating the waveform trend, and $\mathrm{IWLSF}\left(\cdot \right)$ represents IWLSF surface fitting for the data points in brackets.

The DI constructed in this work has three key advantages: (1) The DI demonstrates enhanced noise robustness. The correlation analysis of wavelet-transformed mode shapes at different scales effectively filters noise, making the DI particularly well suited for DIC techniques in mode-shape-based damage identification. (2) During IWLSF, damage peak points are treated as outliers and assigned near-zero weights. This results in a smoother, more accurately fitted trend $WT_{i}^{\mathrm{TR}}$. This means that it can eliminate waveform trend without reducing the degree of damage featured in $WT_{i}^{DO}$ obtained by the difference operation. (3) Noise distribution varies across different intermediate scales, while the theoretical maximum damage feature value remains constant. By accumulating and weighting multiscale data, this DI amplifies the damage feature while simultaneously mitigating noise.

## 3. Validation Using Numerical Simulation

### 3.1. Case Description and Modeling

In order to verify the feasibility and effectiveness of the proposed method, finite element models of plates with two common types of damage—breathing cracks and regional notch damage—were established using ANSYS 2020R2 for numerical simulation. The geometric parameters of the damaged plates, including the length (L), width (W), and thickness (H), and the material parameters, including elastic modulus (E), Poisson’s ratio (ν), and density ($\rho_{0}$), are given in Table 1. The breathing crack was specified by the relative crack depth ζ (ratio of crack depth to plate thickness) and its length was 0.04 m. The regional notch damage was specified by the relative damage length of area ξ (ratio of damage length to plate length) and the depth was 0.00015 m.

**Figure 10:**
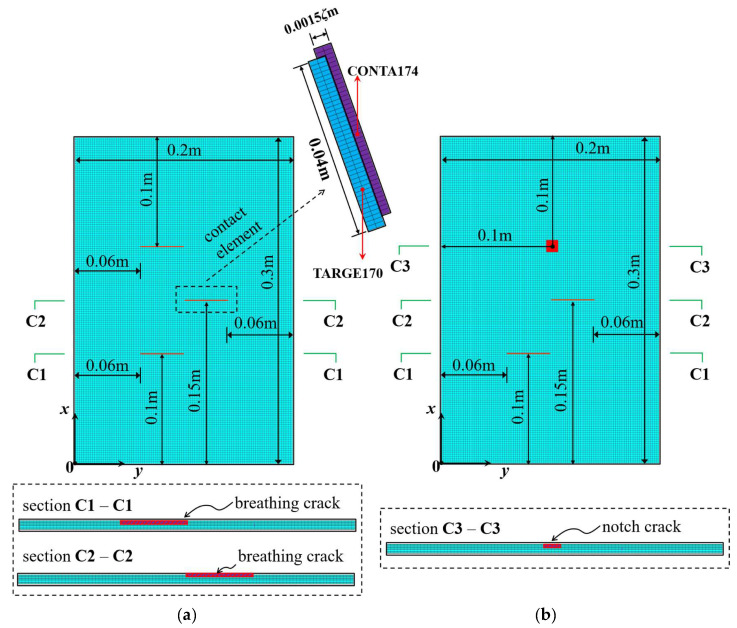
FE model of cantilever plate with multiple damage locations: (**a**) damage case M1 (with three breathing cracks); (**b**) damage case M2 (with two breathing cracks and one notch).

Two multiple-damage cases were set: one was a multi-crack damage case, M1, and the other was a crack and notch damage mixed-damage case, M2. The cantilever plate was established and discretized with eight-node solid elements, as shown in Figure 10. The element size was 0.002 m along the plate length and width direction, and the element size in the plate thickness direction was set according to the damage depth. In this work, the elements TARGE170 and CONTA174 were used to set frictionless standard contact pairs on two opposite crack surfaces. In order to prevent the crack surfaces from penetrating each other, contact stiffness was set on the contact pairs according to the method in references [39,40]. An impact load of 500 N was applied to the plate surface nodes along the plate thickness direction. The displacement responses of the all nodes along the thickness direction of the plate were calculated by ANSYS 2020R2 transient dynamic analysis, and the Rayleigh damping assumption was adopted in the calculation and analysis. The sampling frequency was set to 500 Hz and the sampling time was 3 s. The first-three-order mode shapes of the damaged plates are shown in Table 2. They were obtained by simulating the OMA approach adopted in this work in order to make the simulation more realistic.

Measured dynamic responses inevitably contain noise due to environmental temperature, humidity changes, and equipment limitations. To simulate realistic conditions, Gaussian white noise was added to the extracted mode shapes. Damage detection was then performed using these noise-contaminated mode shapes. The noise-added mode shapes can be expressed as follows [34]:

(22) $$W_{N}\left(x,y\right)=W\left(x,y\right)+\rho \cdot r\cdot w_{rms}$$

where $W_{N}\left(x,y\right)$ is the noise-added mode shape, $W\left(x,y\right)$ is the mode shape without noise, ρ is the white noise intensity, r is a set of random numbers satisfying a normal distribution with a mean of 0 and a variance of 1, and $w_{rms}$ is the root mean square of the mode shape.

### 3.2. Results of Damage Identification

**Figure 11:**
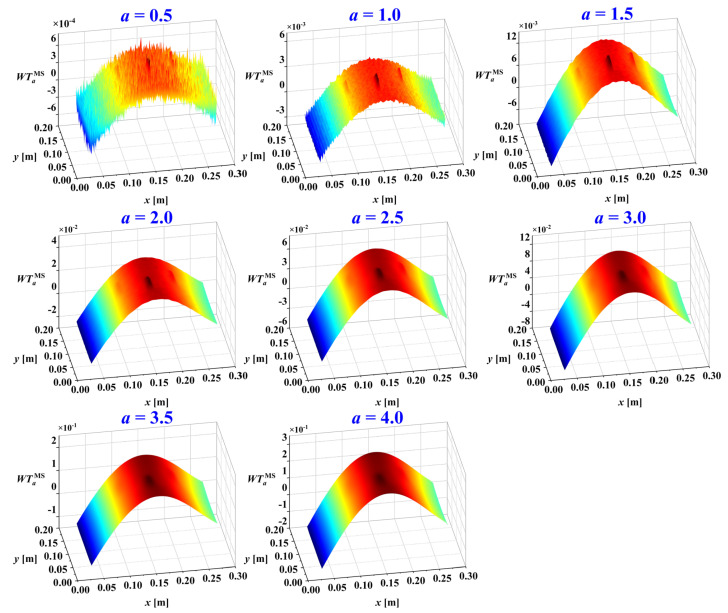
Wavelet coefficients $WT_{a}^{\mathrm{MS}}$ of third mode shape under 8 different scales of M1 ($\rho =1\times 10^{-4}$).

Considering the multi-crack damage case M1, its third mode shape under a noise intensity of $\rho =1\times 10^{-4}$ was used to identify the damage. It was decomposed using 2D-CWT (Mexican-hat wavelet) with scales ranging from *a* = 0.5 to 4.0 in increments of 0.5. The resulting wavelet coefficients, excluding distorted boundary coefficients, are presented in Figure 11. The damage information is obscured by noise at small scales, while the dominant waveform trend at large scales mask its presence, as demonstrated in Figure 11. Furthermore, the damage features are not readily apparent at intermediate scales.

**Figure 12:**
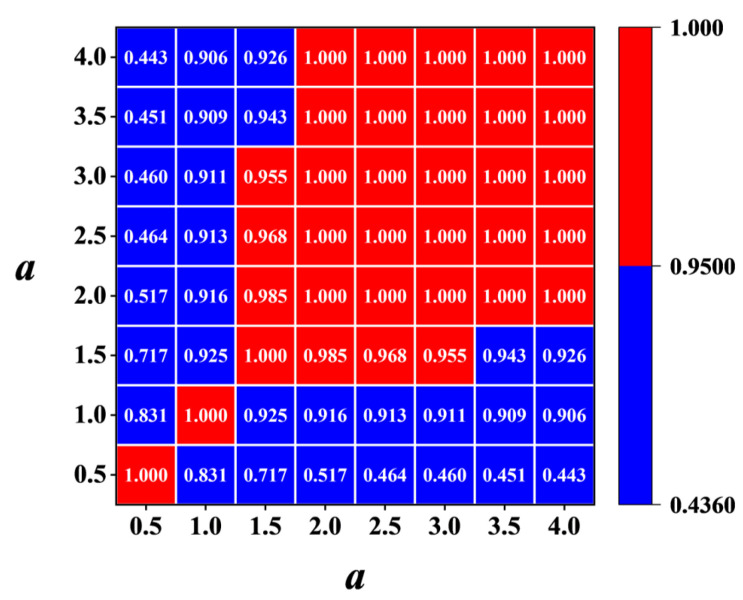
Correlation coefficient *R* between $WT_{a}^{\mathrm{MS}}$ of different scales *a* of damage case M1 ($\rho =1\times 10^{-4}$).

**Figure 13:**
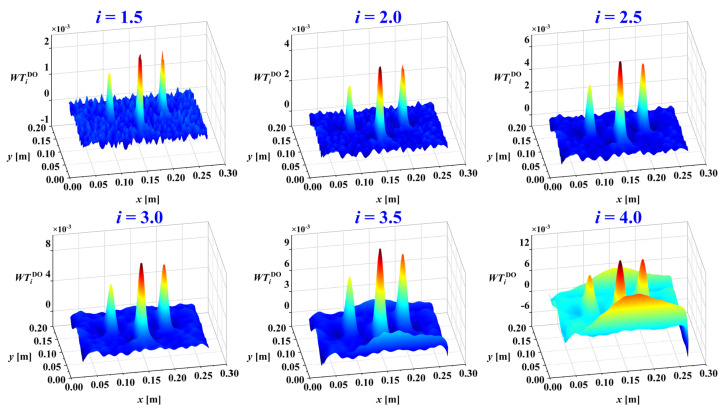
$WT_{i}^{DO}$ of residual scales after noise elimination and trend elimination ($\rho =1\times 10^{-4}$).

**Figure 14:**
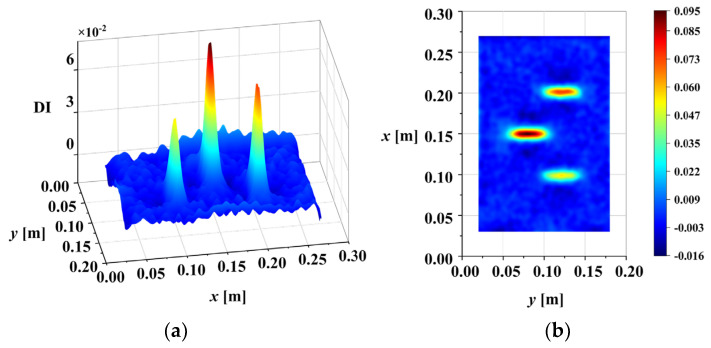
DI maps of third mode shape of M1 ($\rho =1\times 10^{-4}$): (**a**) three-dimensional surface plot; (**b**) X-Y view map.

Figure 12 illustrates the correlation coefficients, R, between these scales, calculated using Equation (11). Lower correlation coefficients indicate that these scales may contain less-reliable damage information due to the influence of noise. Scale information with a correlation coefficient below the threshold $R_{\eta }=0.95$ should be eliminated. Here, the scales 0.5 and 1 are eliminated. After that, IWLSF was performed on the remaining scales, $WT_{i}^{DO}$ was calculated by subtraction, and the results are shown in Figure 13. After removing noise scales and eliminating waveform trends, the abrupt peaks caused by damage are highlighted. Finally, the DI was calculated using Equation (19). The three-dimensional surface plot and X-Y view of the DI map are illustrated in Figure 14.

**Figure 15:**
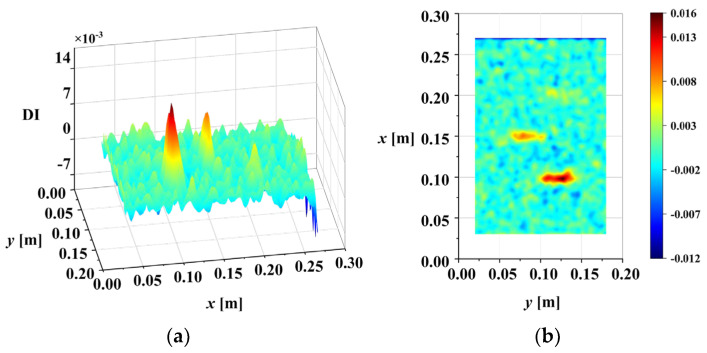
DI map of first mode shape of M1 ($\rho =1\times 10^{-4}$): (**a**) three-dimensional surface plot; (**b**) X-Y view map.

**Figure 16:**
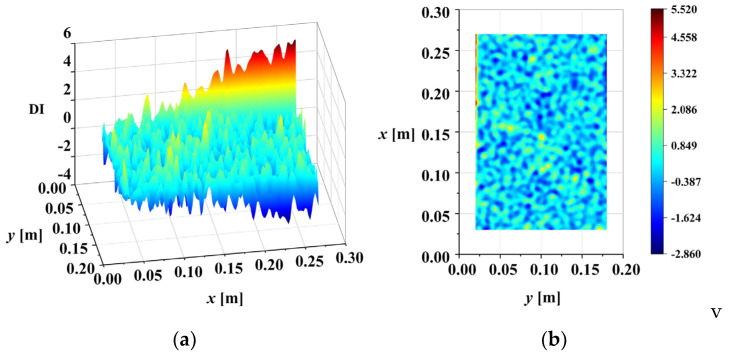
DI map of second mode shape of M1 ($\rho =1\times 10^{-4}$): (**a**) three-dimensional surface plot; (**b**) X-Y view map.

In conclusion, based on the third-order mode shape of M1, the multi-crack damage can be accurately located in the noisy environment ($\rho =1\times 10^{-4}$) using the DI. The first- and second-order mode shapes of M1 in the noise environment ($\rho =1\times 10^{-4}$) were processed according to the above flow, and the DI maps are shown in Figure 15 and Figure 16, respectively. It can be seen from the figure that the damage localization results based on the first bending mode and the second torsional mode are not ideal, especially for the second mode, where there is no damage abrupt peak at all. The reason for this may be that bending mode is more sensitive to crack damage than the torsion mode, and the higher-order mode contains more singular information to reveal damage. Based on the above analysis, the third-order bending mode shape is adopted to identify damage below. Although higher-order mode shapes have potential advantages for damage identification, they are not adopted in this work because they are highly susceptible to noise interference and difficult to measure accurately in real-world conditions.

**Figure 17:**
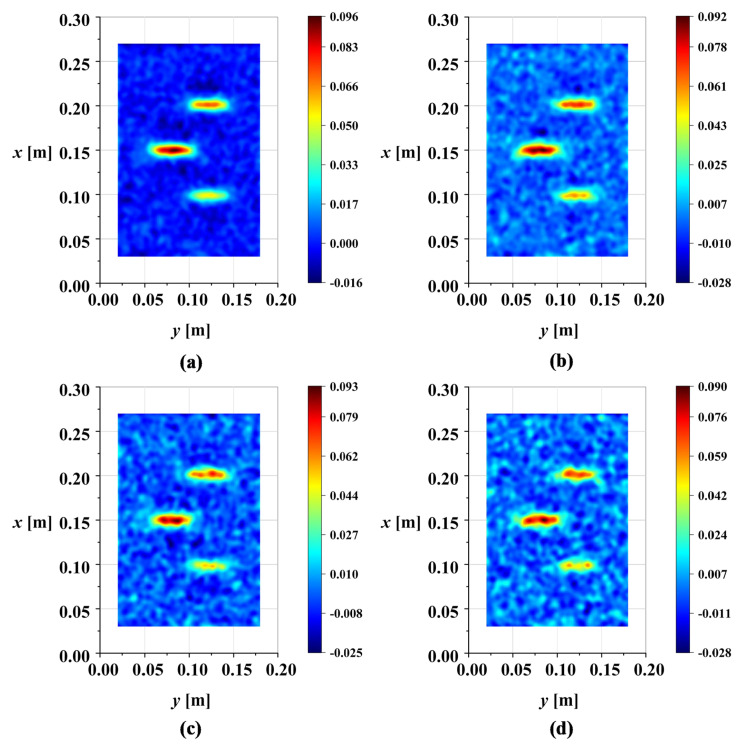
X-Y view of DI maps of M1 in noise environment: (**a**) $\rho =2\times 10^{-4}$; (**b**) $\rho =3\times 10^{-4}$; (**c**) $\rho =4\times 10^{-4}$; (**d**) $\rho =5\times 10^{-4}$.

The above results were obtained under a noise intensity of $\rho =1\times 10^{-4}$. The identification results for M1 under different noise intensities are shown in Figure 17. In order to quantify the noise robustness of the proposed DI, a noise sensitivity analysis of the DI was conducted. A normalized reference damage index function $DIF\left(x,y\right)$ was constructed, with value 1 in a damaged area and value 0 in an intact area, and its expression is as follows:

(23) $$DIF\left(x,y\right)=\left\{\begin{array}{l} 1,\;\mathrm{damaged}\;\mathrm{area}\\ 0,\;\mathrm{intact}\;\mathrm{area} \end{array}\right.$$

A correlation analysis between the $DIF\left(x,y\right)$ and the DI at various noise intensities was conducted using Equation (11). In this analysis, all of the DIs were normalized. A higher correlation coefficient indicates being less sensitive to noise and more robust. The correlation coefficients of damage case M1 under different noise intensities are shown in Table 3. In addition, the CMS2D method was evaluated for comparison. Ideally, the DI should be 1 only at the location of the damage. However, due to the amplification effects of damage identification methods in surrounding areas, this ideal is difficult to achieve, leading to a relatively low correlation. To evaluate the effectiveness of the damage indices under different noise intensities, the effectiveness of the noise-free DI was set to 1. The correlation coefficients of the DIs under noisy conditions were normalized by the noise-free correlation coefficient $R_{0}$, and normalization is expressed as follows:

(24) $$E=\frac{R_{\rho }}{R_{0}}$$

where E represents the normalized effectiveness, $R_{0}$ represents the correlation coefficient under noise-free conditions, and $R_{\rho }$ is the correlation coefficient under a specific noise intensity ρ.

Table 3 demonstrates that the effectiveness of the proposed method can exceed 95% at a noise intensity of $\rho \leq 3\times 10^{-4}$. Therefore, without any additional noise-reduction algorithms, the proposed DI can accurately detect crack locations with a noise intensity of $\rho \leq 3\times 10^{-4}$.

**Figure 18:**
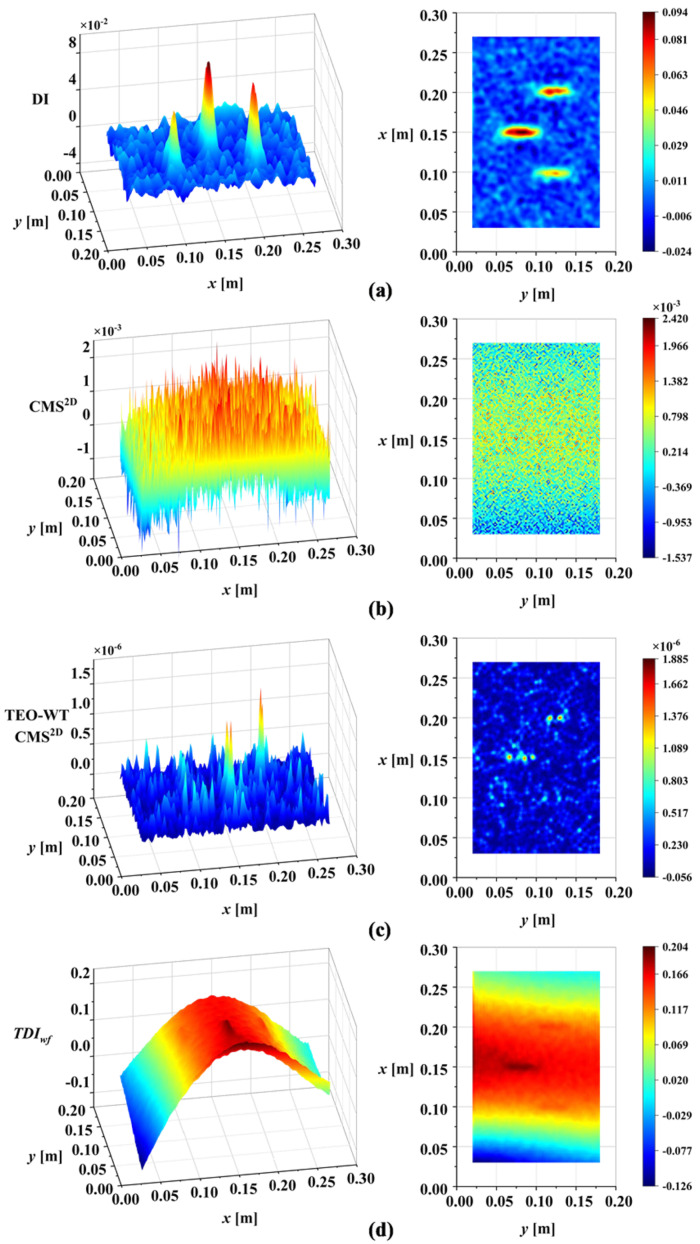
Comparison with existing methods for damage case M1 ($\rho =3\times 10^{-4}$): (**a**) DI proposed in this paper (using third mode shape); (**b**) CMS^2D^ (using third mode shape); (**c**) TEO-WT CMS^2D^ (using third mode shape); (**d**) *TDI_wf_* (using first-three-order mode shapes).

To show the improvement of the proposed DI in this work, the DI was compared with CMS^2D^, TEO-WT CMS^2D^, and *TDI_wf_*. The DI maps of the four methods are presented in Figure 18. Further, to quantify the performance of our method, a generic sensitivity analysis of the DIs to damage was carried out. The sensitivity of the different DIs to damage was discussed by analyzing the correlation between $DIF\left(x,y\right)$ and the DIs established by the different methods. A higher correlation indicated a greater sensitivity to damage, suggesting higher effectiveness in damage identification. For a unified assessment of the four methods, 100 damage detection trials were conducted for each method under random noise of intensity $\rho =3\times 10^{-4}$. The results were then averaged and normalized for sensitivity analysis. Table 4 presents the correlation analysis results between the normalized statistical mean of each method’s DI and the normalized reference index $DIF\left(x,y\right)$.

The proposed DI offers significant improvements over existing wavelet-based methods, as illustrated in Table 4. Specifically, existing methods suffer from several limitations. Although the TEO-WT CMS^2D^ method can identify damage under certain conditions, its reliance on manual wavelet scale selection introduces subjectivity and potential for error. If the wavelet scale adopted is not appropriate, the TEO-WT CMS^2D^ method will fail to detect damage. The *TDI_wf_* method, while incorporating multiple scales, suffers from low noise robustness, requiring additional noise-reduction algorithms. Furthermore, the waveform trend significantly affects its performance, potentially masking damage features. In contrast, the proposed DI demonstrates distinctive features that effectively characterize the presence of damage. By using correlation analysis to determine the wavelet scale, subjectivity is avoided. Based on MIS, the proposed DI separates noise and waveform trends, highlighting damage information and resulting in effective damage identification.

**Figure 19:**
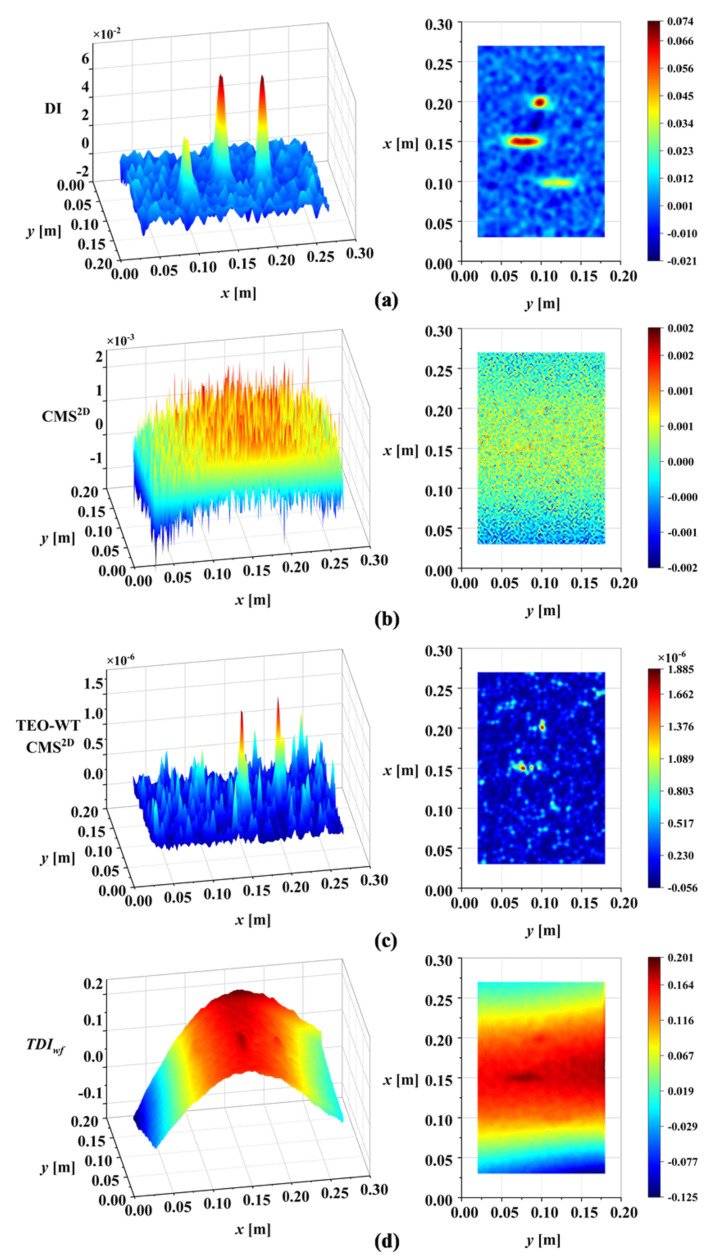
Comparison with existing methods for damage case M2 ($\rho =3\times 10^{-4}$): (**a**) DI proposed in this paper (using third mode shape); (**b**) CMS^2D^ (using third mode shape); (**c**) TEO-WT CMS^2D^ (using third mode shape); (**d**) *TDI_wf_* (using first-three-order mode shapes).

To verify the universality of the proposed DI for different damage types, based on the analysis above, the third-order mode shape of damage case M2 was tested directly under a noise intensity of $\rho =3\times 10^{-4}$. Figure 19a illustrates the DI maps for damage case M2. The results confirm the applicability of the proposed DI to both linear damage (cracks) and regional damage. A comparison with existing methods for damage case M2 is shown in Figure 19. Table 5 presents the sensitivity to mixed damage (damage case M2) of each method, found by correlation analysis.

## 4. Experimental Validation

To verify the practical applicability of the proposed method, damage detection experiments were carried out on an aluminum plate with artificial scratches. The optical dynamic measurement system was used as the measurement technology supporting the damage identification method. The full-field displacement response of the aluminum plate surface was reconstructed by stereo-DIC, and the first-three-order modal parameters of the aluminum plate were calculated. The third-order mode shape was processed by the proposed method to locate the scratch damage.

### 4.1. Experimental Set-Up

**Figure 20:**
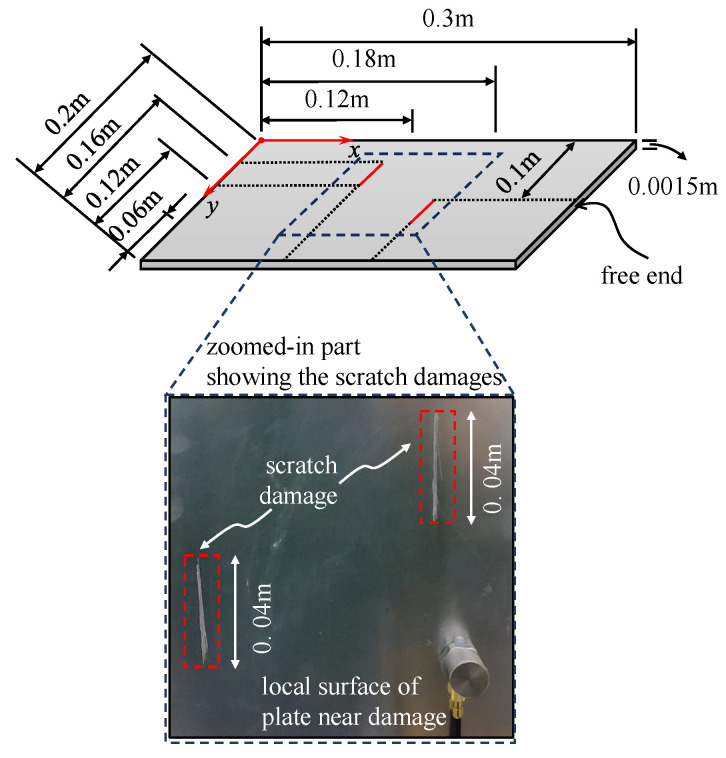
The cantilever aluminum plate and scratch damage positions.

**Figure 21:**
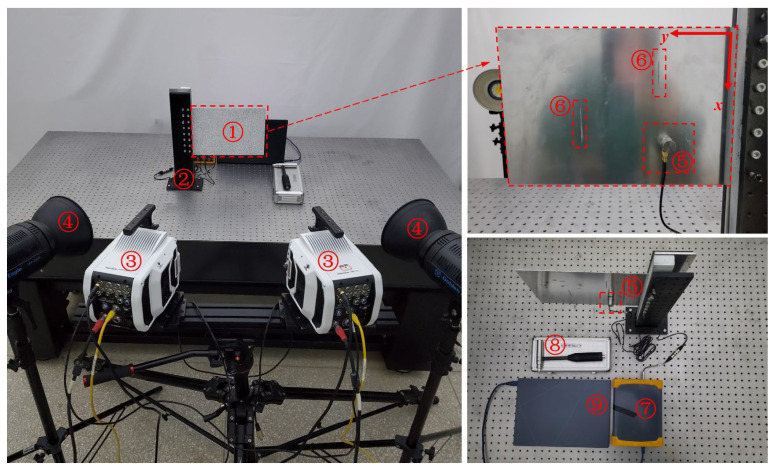
Site layout of experiment (① aluminum plate; ② custom fixture; ③ high-speed cameras; ④ lighting; ⑤ acceleration sensor 1A111E; ⑥ scratch damage; ⑦ signal analyzer DH5928; ⑧ impact hammer; ⑨ laptop).

An aluminum plate of 320 mm in length, 200 mm in width, and 1.5 mm in thickness served as the testing object. One side of the aluminum plate was fixed by a custom fixture to set the cantilever boundary condition. Since the fixture’s clamping length was 20 mm, the cantilever aluminum plate’s effective length was 300 mm. As shown in Figure 20, there were two artificial scratches on the aluminum plate’s surface that were 40 mm long and roughly 1.5 mm wide, parallel to the plate edge. An impact load was applied to the back of the aluminum plate along the thickness direction using an impact hammer to perform the operational modal analysis. The dynamic response of the aluminum plate in the thickness direction was measured using the high-frequency optical measurement system and an accelerometer. The sampling frequency was 1000 Hz, and 6455 speckle images were collected by the high-speed camera at full resolution. Transmitting those images to the computer, the displacement response of each measuring point on the aluminum plate surface was reconstructed from these pictures by Correli^STC^ software (Version 2.1.0.14) based on stereo-DIC. The site layout of the experiment is shown in Figure 21.

### 4.2. Results of Damage Identification

**Figure 22:**
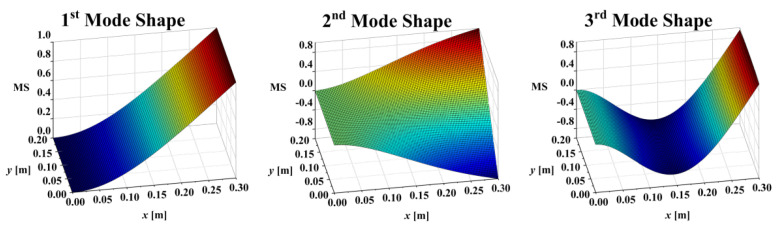
The first three mode shapes of the multi-scratch damaged plate.

**Figure 23:**
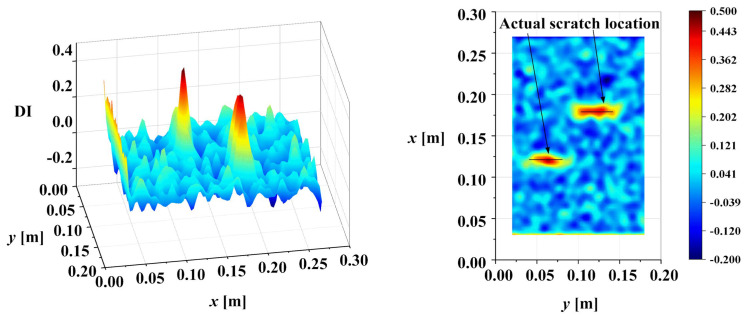
Scratch damage identification results of proposed method.

**Figure 24:**
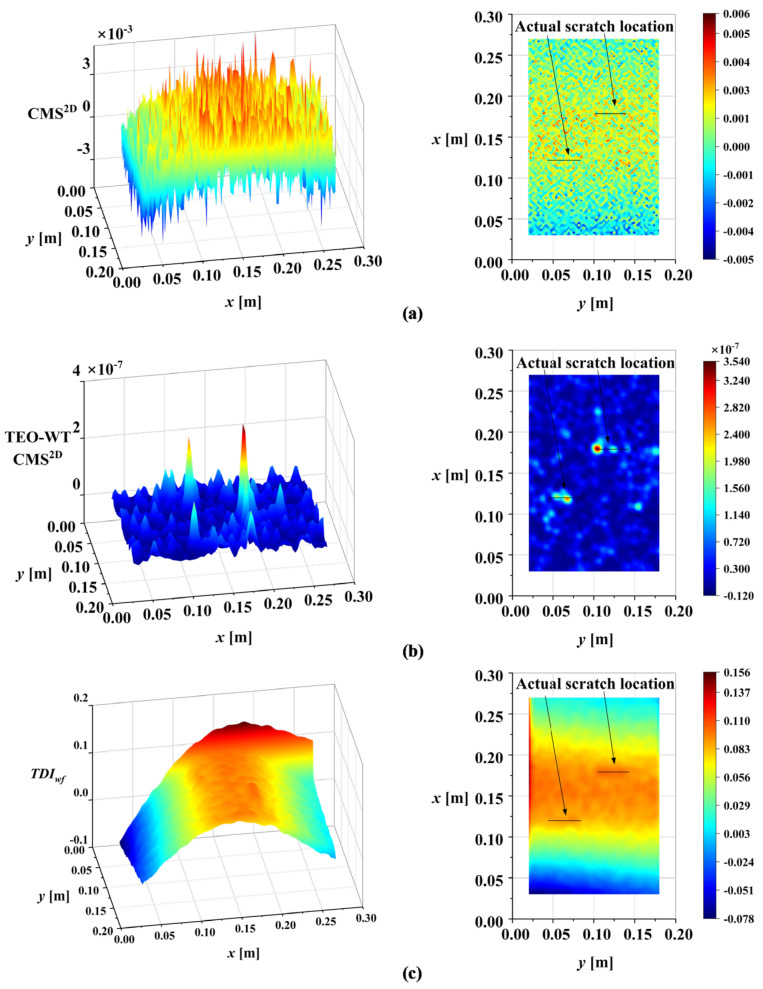
Damage identification results based on existing methods: (**a**) CMS^2D^; (**b**) TEO-WT CMS^2D^; (**c**) *TDI_wf_*.

The mode shapes of the damaged plate were obtained by OMA, as Figure 22 illustrates. Based on the conclusions of the numerical simulation, only the third-order bending mode of the scratched cantilever aluminum plate was analyzed. The damage identification result obtained by using the proposed method in this paper after scale correlation filtering, waveform trend elimination, and multiscale accumulation is shown in Figure 23. The abrupt changes caused by damage can be clearly observed in Figure 23. The solid black line in the figure indicates the actual scratch position. It is worth noting that there were maxima or minima at the boundaries of the experimental results compared with the numerical simulation results. Because the number of measuring points in the experimental results was less than that in the numerical simulation, the distortion coefficients at the boundary were not completely removed. This reflects the great advantage of optical dynamic measurement in obtaining structural mode shapes due to its aerial high-resolution measurement. It can improve the measurement point density compared with traditional contact measurement technology, contributing to reducing the influence of distortion at the boundary. In addition, existing methods were used to identify the damage to show the superiority of the proposed method in contrast. The results of the existing methods are shown in Figure 24. To quantify the effectiveness of the proposed method, a correlation analysis for the experimental results based on various methods with $DIF\left(x,y\right)$ was performed. The results are shown in Table 6.

## 5. Conclusions

Based on the optical dynamic measurement system and vibration-based damage identification theory, a baseline-free damage identification method with high accuracy and noise robustness is proposed in this paper. The reliability and practical applicability of the method were verified by numerical simulation and laboratory experiments of damage detection for plate-like structures. The following conclusions can be drawn:

(1) The mode shape is a modal parameter containing abundant dynamic information because of its spatial distribution characteristics. It is very beneficial to develop the acquisition and processing of high-resolution mode shapes for structural damage identification.

(2) Compared with torsional mode shapes, bending mode shapes are more sensitive to crack damage in plate-like structures. Compared with lower-order modes, higher-order modes contain more spatial information that can reveal damage locations.

(3) CMS^2D^ is sensitive to both damage and noise, requiring additional noise-reduction algorithms for robust damage identification. The proposed method does not have this limitation.

(4) With appropriate wavelet scale selection, TEO-WT CMS^2D^ can achieve effective damage identification. Developing an adaptive wavelet scale selection method for TEO-WT CMS^2D^ would further enhance its effectiveness.

(5) The DI proposed in this paper can accurately identify multiple sites of damage (micro-crack damage with *ζ =* 10% and minor area damage with *ξ =* 5%) in noisy environments with a noise intensity of $\rho \leq 3\times 10^{-4}$. It can not only highlight damage but also suppress noise without additional noise-reduction algorithms.

(6) The amplification effect is obvious in linear damage identification. The intact region near the damaged site often presents larger singular values which affect the identification of damage close to the location. Future studies can address this amplification effect and improve correlation with the normalized reference index $DIF\left(x,y\right)$.

## Figures and Tables

**Table 1:** Parameters of damaged cantilever plate model.

Type	Ε	$\rho_{0}$	ν	L	W	H	ζ	ξ
Value	68	2760	30%	300	200	1.5	10%	5%
Unit	GPa	kg/m^3^	1	mm	mm	mm	1	1

**Table 2:** The first-three-order mode shapes of the damaged plates.

Damage Case	Mode Shape
M1	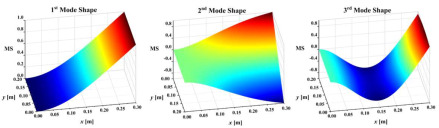
M2	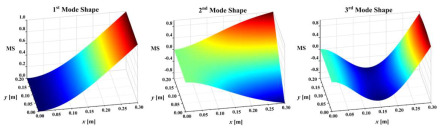

**Table 3:** Effectiveness of DI under different noise intensities (damage case M1).

ρ	DI		CMS^2D^	
	R (%)	Effectiveness (%)	R (%)	Effectiveness (%)
0	44.43	1	7.29	1
1 × 10^−4^	44.14	99.35	5.47	75.03
2 × 10^−4^	43.86	98.72	2.54	34.84
3 × 10^−4^	43.02	96.83	2.47	33.88
4 × 10^−4^	41.65	93.74	1.01	13.85
5 × 10^−4^	38.29	86.18	0.47	6.45

**Table 4:** Sensitivity to damage of various methods for damage case M1 ($\rho =3\times 10^{-4}$).

Damage Index	DI	CMS^2D^	TEO-WT CMS^2D^	*TDI_wf_*
Effectiveness (%)	43.02	4.93	20.34	7.52

**Table 5:** Sensitivity to damage of various methods for damage case M2 ($\rho =3\times 10^{-4}$).

Damage Index	DI	CMS^2D^	TEO-WT CMS^2D^	*TDI_wf_*
Effectiveness (%)	39.72	2.57	23.61	5.94

**Table 6:** Sensitivity to damage of various methods for multiple scratches.

Damage Index	DI	CMS^2D^	TEO-WT CMS^2D^	*TDI_wf_*
Effectiveness (%)	37.21	4.93	27.14	8.76

## Data Availability

All data generated or analyzed during this study are included in this published article.

## Abbreviations

The following abbreviations are used in this manuscript:

MIS	Multicomponent information separation
DI	Damage indicator
2D-CWT	Two-dimensional continuous wavelet transform
SCA	Scale correlation analysis
IWLSF	Iterative weighted least squares fitting
LSF	Least squares fitting
CMS^2D^	Two-dimensional curvature mode shape
TEO-WT CMS^2D^	Two-dimensional curvature mode shape with TEO energy operator and wavelet transform
*TDI_wf_*	Total Damage Index with wavelet
DIC	Digital image correlation
OMA	Operating modal analysis
LSCF	Least Squares Complex Frequency Domain
